# Methyl 5-[*N*,*N*-bis­(methoxy­carbonyl­meth­yl)amino]-4-cyano-2-methoxy­carbonyl-3-thio­phene­ethan­o­ate

**DOI:** 10.1107/S1600536808028699

**Published:** 2008-09-13

**Authors:** Qiang Wang, Zhong-Shu Li, Bai-Wang Sun

**Affiliations:** a Ordered Matter Science Research Center, College of Chemistry and Chemical Engineering, Southeast University, Nanjing 210096, People’s Republic of China

## Abstract

In the title compound, C_16_H_18_N_2_O_8_S, derived from ranelic acid, there is a highly substituted thio­phene ring. The crystal structure involves inter­molecular C—H⋯O and C—H⋯S hydrogen bonds.

## Related literature

For related literature, see: Bonnelye *et al.* (2008 ▶); Fonseca (2008 ▶).
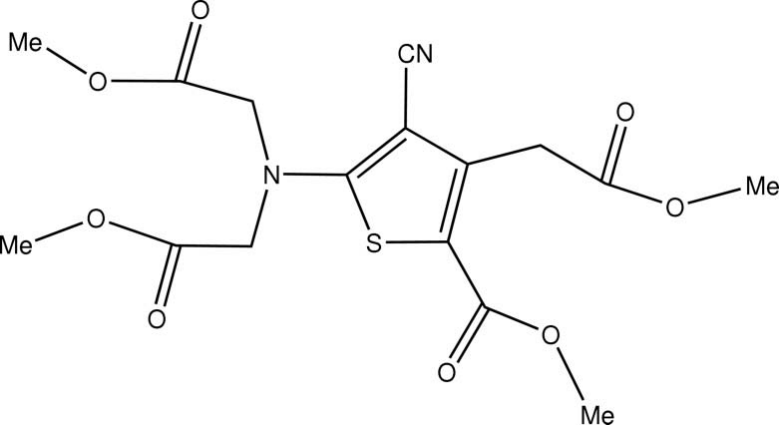


## Experimental

### 

#### Crystal data

C_16_H_18_N_2_O_8_S
*M* *_r_* = 398.38
Triclinic, 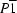

*a* = 9.7164 (19) Å
*b* = 9.790 (2) Å
*c* = 10.170 (2) Å
α = 98.05 (3)°
β = 96.71 (3)°
γ = 95.81 (3)°
*V* = 944.5 (3) Å^3^
*Z* = 2
Mo *K*α radiation
μ = 0.22 mm^−1^
*T* = 293 (2) K
0.25 × 0.20 × 0.18 mm

#### Data collection

Rigaku SCXmini diffractometer
Absorption correction: multi-scan (*CrystalClear*; Rigaku, 2005 ▶) *T*_min_ = 0.942, *T*_max_ = 0.988
9976 measured reflections
4313 independent reflections
3561 reflections with *I* > 2σ(*I*)
*R*_int_ = 0.027

#### Refinement

*R*[*F* ^2^ > 2σ(*F* ^2^)] = 0.048
*wR*(*F* ^2^) = 0.130
*S* = 1.03
4313 reflections
246 parameters
H atoms treated by a mixture of independent and constrained refinement
Δρ_max_ = 0.33 e Å^−3^
Δρ_min_ = −0.38 e Å^−3^

### 

Data collection: *CrystalClear* (Rigaku, 2005 ▶); cell refinement: *CrystalClear*; data reduction: *CrystalClear*; program(s) used to solve structure: *SHELXS97* (Sheldrick, 2008 ▶); program(s) used to refine structure: *SHELXL97* (Sheldrick,2008 ▶); molecular graphics: *SHELXTL* (Sheldrick, 2008 ▶); software used to prepare material for publication: *SHELXL97*.

## Supplementary Material

Crystal structure: contains datablocks I, global. DOI: 10.1107/S1600536808028699/sg2258sup1.cif

Structure factors: contains datablocks I. DOI: 10.1107/S1600536808028699/sg2258Isup2.hkl

Additional supplementary materials:  crystallographic information; 3D view; checkCIF report

## Figures and Tables

**Table 1:** Hydrogen-bond geometry (Å, °)

*D*—H⋯*A*	*D*—H	H⋯*A*	*D*⋯*A*	*D*—H⋯*A*
C3—H3*A*⋯O3^i^	0.97	2.51	3.355 (3)	146
C16—H16*A*⋯O5^ii^	0.96	2.57	3.421 (3)	148
C16—H16*C*⋯S^ii^	0.96	2.87	3.727 (3)	149

Symmetry codes: (i) 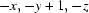; (ii) 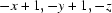.

**Fig. 1.**
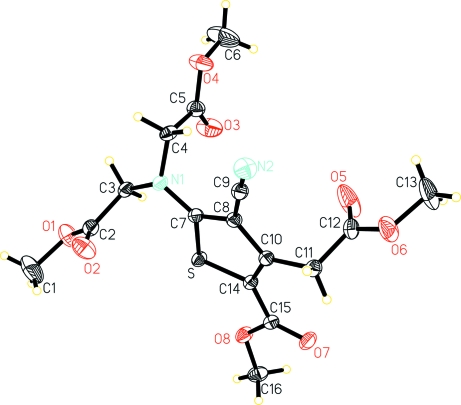
The molecular structure of the title molecule, with the atom-numbering scheme. Displacement ellipsoids are drawn at the 30% probability level.

**Fig. 2.**
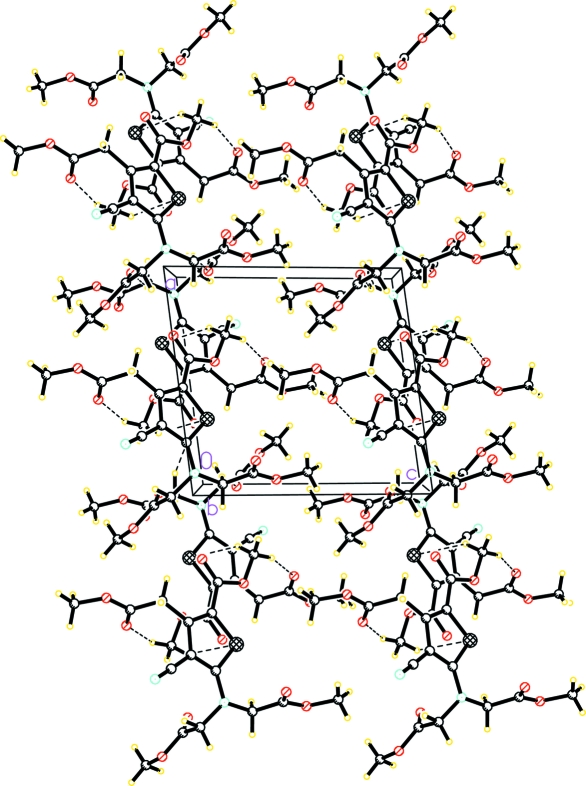
Crystal packing of the compound (I) viewed along the b axis. Hydrogen bonds are shown as dashed lines.

## supplementary crystallographic information

### Comment

The title compound, (I), C_16_H_18_O_8_N_2_S, is an important intermediate in the synthesis of strontium ranelate, a medicine for the treatment of osteoporosis (Bonnelye *et al.*, 2008; Fonseca, 2008). Strontium ranelate is composed of two stable strontium ions combined with the anion of organic ranelic acid. The ranelic acid is a carrier that makes the treatment palatable, and the strontium is the active component with regard to the bone. We report here the crystal structure of the title compound. The molecular structure of (I) is shown in Fig. 1 and geometric parameters are given in Table 1. In the crystal (Fig. 2 and Table 2), there are intermolecular C—H···O and C—H···S hydrogen bonds leading to a one-dimensional structure.

### Experimental

All chemicals used (reagent grade) were commercially available. Methyl 5-[bis(methoxycarbonylmethyl)amino]-4-cyano-3-(methoxycarbonyl)-2-thiophene- carboxylate (0.426 g, 0.1 mmol) was added to a solution containing ethanol (8 ml) and acetone (4 ml). The mixture was stirred at room temperature for 10 min and then filtered off. Colorless crystals suitable for X-ray analysis were obtained by slow evaporation at room temperature over several days.

### Refinement

All H atoms attached to C and N atom were fixed geometrically and treated as riding with C—H = 0.97 Å with *U*_iso_(H) =1.2*U*_eq_(C).

### Crystal data

**Table:**

C_16_H_18_N_2_O_8_S	*Z* = 2
*M**_r_* = 398.38	*F*(000) = 416
Triclinic, *P*1	*D*_x_ = 1.401 Mg m^−^^3^
Hall symbol: -P 1	Mo *K*α radiation, λ = 0.71073 Å
*a* = 9.7164 (19) Å	Cell parameters from 6733 reflections
*b* = 9.790 (2) Å	θ = 3.3–27.3°
*c* = 10.170 (2) Å	µ = 0.22 mm^−^^1^
α = 98.05 (3)°	*T* = 293 K
β = 96.71 (3)°	Prism, colorless
γ = 95.81 (3)°	0.25 × 0.20 × 0.18 mm
*V* = 944.5 (3) Å^3^	

### Data collection

**Table:**

Rigaku SCXmini diffractometer	4313 independent reflections
Radiation source: fine-focus sealed tube	3561 reflections with *I* > 2σ(*I*)
graphite	*R*_int_ = 0.027
Detector resolution: 8.192 pixels mm^-1^	θ_max_ = 27.5°, θ_min_ = 3.1°
ω scans	*h* = −12→12
Absorption correction: multi-scan (CrystalClear; Rigaku, 2005)	*k* = −12→12
*T*_min_ = 0.942, *T*_max_ = 0.988	*l* = −13→13
9976 measured reflections	

### Refinement

**Table:**

Refinement on *F*^2^	Primary atom site location: structure-invariant direct methods
Least-squares matrix: full	Secondary atom site location: difference Fourier map
*R*[*F*^2^ > 2σ(*F*^2^)] = 0.048	Hydrogen site location: inferred from neighbouring sites
*wR*(*F*^2^) = 0.130	H atoms treated by a mixture of independent and constrained refinement
*S* = 1.03	*w* = 1/[σ^2^(*F*_o_^2^) + (0.062*P*)^2^ + 0.411*P*] where *P* = (*F*_o_^2^ + 2*F*_c_^2^)/3
4313 reflections	(Δ/σ)_max_ < 0.001
246 parameters	Δρ_max_ = 0.33 e Å^−^^3^
0 restraints	Δρ_min_ = −0.38 e Å^−^^3^

### Special details

**Table:**

Geometry. All e.s.d.'s (except the e.s.d. in the dihedral angle between two l.s. planes) are estimated using the full covariance matrix. The cell e.s.d.'s are taken into account individually in the estimation of e.s.d.'s in distances, angles and torsion angles; correlations between e.s.d.'s in cell parameters are only used when they are defined by crystal symmetry. An approximate (isotropic) treatment of cell e.s.d.'s is used for estimating e.s.d.'s involving l.s. planes.
Refinement. Refinement of *F*^2^ against ALL reflections. The weighted *R*-factor *wR* and goodness of fit *S* are based on *F*^2^, conventional *R*-factors *R* are based on *F*, with *F* set to zero for negative *F*^2^. The threshold expression of *F*^2^ > σ(*F*^2^) is used only for calculating *R*-factors(gt) *etc*. and is not relevant to the choice of reflections for refinement. *R*-factors based on *F*^2^ are statistically about twice as large as those based on *F*, and *R*- factors based on ALL data will be even larger.

### Fractional atomic coordinates and isotropic or equivalent isotropic displacement parameters (Å^2^)

**Table:**

	*x*	*y*	*z*	*U*_iso_*/*U*_eq_	
C1	0.0925 (5)	0.8277 (4)	0.4871 (3)	0.0967 (13)	
H1A	0.165 (3)	0.894 (3)	0.4933 (6)	0.145*	
H1B	0.116 (3)	0.7635 (17)	0.5387 (14)	0.145*	
H1C	0.018 (2)	0.866 (3)	0.5154 (12)	0.145*	
C2	0.0887 (2)	0.8372 (2)	0.2560 (2)	0.0414 (5)	
C3	0.03705 (19)	0.7542 (2)	0.11943 (19)	0.0374 (4)	
H3A	0.0661	0.6620	0.1164	0.045*	
H3B	−0.0642	0.7441	0.1059	0.045*	
C4	−0.01502 (19)	0.8243 (2)	−0.1025 (2)	0.0366 (4)	
H4A	0.0231	0.8874	−0.1582	0.044*	
H4B	−0.0962	0.8606	−0.0702	0.044*	
C5	−0.0589 (2)	0.6829 (2)	−0.1857 (2)	0.0427 (5)	
C6	−0.2176 (5)	0.5578 (4)	−0.3673 (4)	0.1086 (14)	
H6A	−0.2891	0.5740	−0.4348	0.163*	
H6B	−0.2567	0.4957	−0.3126	0.163*	
H6C	−0.1448	0.5171	−0.4094	0.163*	
C7	0.22416 (18)	0.80692 (18)	−0.00849 (18)	0.0312 (4)	
C8	0.30195 (18)	0.86329 (19)	−0.09923 (18)	0.0321 (4)	
C9	0.2567 (2)	0.9617 (2)	−0.18082 (19)	0.0369 (4)	
C10	0.43987 (18)	0.82416 (19)	−0.09451 (18)	0.0335 (4)	
C11	0.5374 (2)	0.8694 (2)	−0.18941 (19)	0.0404 (4)	
H11A	0.5404	0.9690	−0.1876	0.048*	
H11B	0.6307	0.8498	−0.1592	0.048*	
C12	0.4935 (2)	0.7974 (2)	−0.3310 (2)	0.0446 (5)	
C13	0.5241 (4)	0.8027 (4)	−0.5571 (3)	0.0940 (11)	
H13A	0.5736	0.8606	−0.6092	0.141*	
H13B	0.4257	0.7956	−0.5864	0.141*	
H13C	0.5541	0.7119	−0.5686	0.141*	
C14	0.46807 (18)	0.7442 (2)	0.00257 (18)	0.0335 (4)	
C15	0.59750 (19)	0.6869 (2)	0.04245 (19)	0.0360 (4)	
C16	0.7100 (2)	0.5700 (2)	0.2060 (2)	0.0501 (5)	
H16A	0.6891	0.5245	0.2802	0.075*	
H16B	0.7815	0.6466	0.2366	0.075*	
H16C	0.7418	0.5053	0.1396	0.075*	
O1	0.0564 (2)	0.76176 (18)	0.34904 (16)	0.0637 (5)	
O2	0.1471 (2)	0.95261 (18)	0.27677 (18)	0.0673 (5)	
O3	−0.0096 (2)	0.57999 (18)	−0.1647 (2)	0.0719 (6)	
O4	−0.15997 (19)	0.68911 (18)	−0.28405 (17)	0.0626 (5)	
O5	0.4141 (3)	0.6943 (2)	−0.3638 (2)	0.1020 (9)	
O6	0.5525 (2)	0.8633 (2)	−0.41698 (17)	0.0695 (5)	
O7	0.70142 (14)	0.69773 (18)	−0.01103 (16)	0.0520 (4)	
O8	0.58547 (14)	0.62078 (16)	0.14789 (15)	0.0438 (3)	
N1	0.08891 (15)	0.81812 (17)	0.01103 (16)	0.0346 (3)	
N2	0.2253 (2)	1.0438 (2)	−0.2444 (2)	0.0535 (5)	
S	0.32536 (5)	0.71175 (5)	0.08736 (5)	0.03457 (14)	

### Atomic displacement parameters (Å^2^)

**Table:**

	*U*^11^	*U*^22^	*U*^33^	*U*^12^	*U*^13^	*U*^23^
C1	0.151 (4)	0.103 (3)	0.0364 (14)	0.029 (2)	0.0078 (17)	0.0058 (15)
C2	0.0371 (10)	0.0491 (12)	0.0399 (11)	0.0130 (9)	0.0055 (8)	0.0071 (9)
C3	0.0266 (9)	0.0471 (11)	0.0394 (10)	0.0020 (7)	0.0078 (7)	0.0087 (8)
C4	0.0280 (9)	0.0392 (10)	0.0447 (11)	0.0078 (7)	0.0016 (8)	0.0136 (8)
C5	0.0412 (11)	0.0444 (11)	0.0418 (11)	0.0083 (9)	−0.0004 (8)	0.0071 (9)
C6	0.132 (3)	0.080 (2)	0.088 (2)	0.010 (2)	−0.054 (2)	−0.0192 (19)
C7	0.0275 (8)	0.0346 (9)	0.0308 (9)	0.0026 (7)	0.0019 (7)	0.0052 (7)
C8	0.0301 (9)	0.0364 (9)	0.0297 (9)	0.0015 (7)	0.0031 (7)	0.0069 (7)
C9	0.0335 (9)	0.0429 (10)	0.0348 (10)	0.0016 (8)	0.0064 (7)	0.0084 (8)
C10	0.0292 (9)	0.0388 (10)	0.0312 (9)	−0.0010 (7)	0.0047 (7)	0.0039 (8)
C11	0.0329 (10)	0.0530 (12)	0.0366 (10)	−0.0014 (8)	0.0081 (8)	0.0128 (9)
C12	0.0494 (12)	0.0491 (12)	0.0391 (11)	0.0046 (10)	0.0171 (9)	0.0112 (9)
C13	0.137 (3)	0.106 (3)	0.0417 (15)	−0.002 (2)	0.0369 (17)	0.0125 (16)
C14	0.0259 (8)	0.0432 (10)	0.0318 (9)	0.0027 (7)	0.0055 (7)	0.0068 (8)
C15	0.0280 (9)	0.0429 (10)	0.0358 (10)	0.0015 (7)	0.0030 (7)	0.0045 (8)
C16	0.0396 (11)	0.0547 (13)	0.0573 (13)	0.0102 (9)	−0.0046 (10)	0.0188 (11)
O1	0.0920 (13)	0.0632 (11)	0.0376 (8)	0.0077 (9)	0.0124 (8)	0.0119 (8)
O2	0.0858 (13)	0.0538 (10)	0.0547 (10)	−0.0060 (9)	−0.0015 (9)	0.0011 (8)
O3	0.0808 (13)	0.0454 (9)	0.0806 (13)	0.0218 (9)	−0.0235 (10)	−0.0024 (9)
O4	0.0680 (11)	0.0608 (10)	0.0513 (10)	0.0112 (8)	−0.0206 (8)	0.0029 (8)
O5	0.161 (2)	0.0805 (14)	0.0505 (11)	−0.0555 (15)	0.0369 (13)	−0.0065 (10)
O6	0.0858 (13)	0.0810 (12)	0.0413 (9)	−0.0148 (10)	0.0204 (9)	0.0163 (9)
O7	0.0305 (7)	0.0792 (11)	0.0519 (9)	0.0127 (7)	0.0118 (6)	0.0202 (8)
O8	0.0322 (7)	0.0563 (9)	0.0478 (8)	0.0104 (6)	0.0060 (6)	0.0207 (7)
N1	0.0255 (7)	0.0430 (9)	0.0366 (8)	0.0047 (6)	0.0049 (6)	0.0094 (7)
N2	0.0579 (12)	0.0532 (11)	0.0536 (11)	0.0069 (9)	0.0061 (9)	0.0237 (9)
S	0.0279 (2)	0.0437 (3)	0.0358 (3)	0.00654 (18)	0.00741 (17)	0.0147 (2)

### Geometric parameters (Å, °)

**Table:**

C1—O1	1.448 (3)	C8—C9	1.427 (3)
C1—H1A	0.9033	C8—C10	1.428 (3)
C1—H1B	0.9033	C9—N2	1.143 (3)
C1—H1C	0.9033	C10—C14	1.363 (3)
C2—O2	1.191 (3)	C10—C11	1.508 (3)
C2—O1	1.325 (3)	C11—C12	1.504 (3)
C2—C3	1.510 (3)	C11—H11A	0.9700
C3—N1	1.456 (2)	C11—H11B	0.9700
C3—H3A	0.9700	C12—O5	1.189 (3)
C3—H3B	0.9700	C12—O6	1.308 (3)
C4—N1	1.455 (2)	C13—O6	1.448 (3)
C4—C5	1.511 (3)	C13—H13A	0.9600
C4—H4A	0.9700	C13—H13B	0.9600
C4—H4B	0.9700	C13—H13C	0.9600
C5—O3	1.193 (3)	C14—C15	1.466 (3)
C5—O4	1.330 (2)	C14—S	1.7420 (18)
C6—O4	1.457 (3)	C15—O7	1.204 (2)
C6—H6A	0.9600	C15—O8	1.338 (2)
C6—H6B	0.9600	C16—O8	1.448 (2)
C6—H6C	0.9600	C16—H16A	0.9600
C7—N1	1.365 (2)	C16—H16B	0.9600
C7—C8	1.396 (2)	C16—H16C	0.9600
C7—S	1.7323 (19)		
			
O1—C1—H1A	109.5	C14—C10—C11	126.48 (17)
O1—C1—H1B	109.5	C8—C10—C11	121.39 (17)
H1A—C1—H1B	109.5	C12—C11—C10	112.45 (16)
O1—C1—H1C	109.5	C12—C11—H11A	109.1
H1A—C1—H1C	109.5	C10—C11—H11A	109.1
H1B—C1—H1C	109.5	C12—C11—H11B	109.1
O2—C2—O1	125.5 (2)	C10—C11—H11B	109.1
O2—C2—C3	125.5 (2)	H11A—C11—H11B	107.8
O1—C2—C3	108.99 (18)	O5—C12—O6	122.9 (2)
N1—C3—C2	112.93 (16)	O5—C12—C11	125.5 (2)
N1—C3—H3A	109.0	O6—C12—C11	111.64 (19)
C2—C3—H3A	109.0	O6—C13—H13A	109.5
N1—C3—H3B	109.0	O6—C13—H13B	109.5
C2—C3—H3B	109.0	H13A—C13—H13B	109.5
H3A—C3—H3B	107.8	O6—C13—H13C	109.5
N1—C4—C5	111.64 (16)	H13A—C13—H13C	109.5
N1—C4—H4A	109.3	H13B—C13—H13C	109.5
C5—C4—H4A	109.3	C10—C14—C15	129.21 (17)
N1—C4—H4B	109.3	C10—C14—S	112.01 (14)
C5—C4—H4B	109.3	C15—C14—S	118.78 (14)
H4A—C4—H4B	108.0	O7—C15—O8	124.03 (18)
O3—C5—O4	125.0 (2)	O7—C15—C14	125.19 (18)
O3—C5—C4	124.21 (19)	O8—C15—C14	110.78 (16)
O4—C5—C4	110.76 (17)	O8—C16—H16A	109.5
O4—C6—H6A	109.5	O8—C16—H16B	109.5
O4—C6—H6B	109.5	H16A—C16—H16B	109.5
H6A—C6—H6B	109.5	O8—C16—H16C	109.5
O4—C6—H6C	109.5	H16A—C16—H16C	109.5
H6A—C6—H6C	109.5	H16B—C16—H16C	109.5
H6B—C6—H6C	109.5	C2—O1—C1	116.7 (2)
N1—C7—C8	129.48 (17)	C5—O4—C6	116.3 (2)
N1—C7—S	120.44 (14)	C12—O6—C13	117.7 (2)
C8—C7—S	110.07 (13)	C15—O8—C16	116.74 (16)
C7—C8—C9	124.59 (17)	C7—N1—C4	119.77 (16)
C7—C8—C10	113.62 (16)	C7—N1—C3	117.45 (15)
C9—C8—C10	121.51 (16)	C4—N1—C3	115.49 (15)
N2—C9—C8	177.3 (2)	C7—S—C14	92.11 (9)
C14—C10—C8	112.12 (16)		
			
O2—C2—C3—N1	11.3 (3)	S—C14—C15—O7	177.27 (17)
O1—C2—C3—N1	−169.76 (17)	C10—C14—C15—O8	176.21 (19)
N1—C4—C5—O3	−3.4 (3)	S—C14—C15—O8	−3.1 (2)
N1—C4—C5—O4	176.17 (17)	O2—C2—O1—C1	1.7 (4)
N1—C7—C8—C9	8.5 (3)	C3—C2—O1—C1	−177.2 (2)
S—C7—C8—C9	−171.41 (15)	O3—C5—O4—C6	3.0 (4)
N1—C7—C8—C10	−177.50 (17)	C4—C5—O4—C6	−176.6 (3)
S—C7—C8—C10	2.6 (2)	O5—C12—O6—C13	−3.2 (4)
C7—C8—C9—N2	129 (5)	C11—C12—O6—C13	177.7 (2)
C10—C8—C9—N2	−45 (5)	O7—C15—O8—C16	5.2 (3)
C7—C8—C10—C14	−2.7 (2)	C14—C15—O8—C16	−174.41 (17)
C9—C8—C10—C14	171.57 (17)	C8—C7—N1—C4	34.7 (3)
C7—C8—C10—C11	177.14 (16)	S—C7—N1—C4	−145.47 (15)
C9—C8—C10—C11	−8.6 (3)	C8—C7—N1—C3	−176.48 (18)
C14—C10—C11—C12	109.2 (2)	S—C7—N1—C3	3.4 (2)
C8—C10—C11—C12	−70.6 (2)	C5—C4—N1—C7	76.6 (2)
C10—C11—C12—O5	−17.2 (4)	C5—C4—N1—C3	−72.8 (2)
C10—C11—C12—O6	161.94 (19)	C2—C3—N1—C7	76.1 (2)
C8—C10—C14—C15	−177.92 (18)	C2—C3—N1—C4	−133.71 (17)
C11—C10—C14—C15	2.3 (3)	N1—C7—S—C14	178.60 (15)
C8—C10—C14—S	1.4 (2)	C8—C7—S—C14	−1.50 (14)
C11—C10—C14—S	−178.35 (15)	C10—C14—S—C7	0.03 (15)
C10—C14—C15—O7	−3.4 (3)	C15—C14—S—C7	179.46 (15)

### Hydrogen-bond geometry (Å, °)

**Table:**

*D*—H···*A*	*D*—H	H···*A*	*D*···*A*	*D*—H···*A*
C3—H3A···O3^i^	0.97	2.51	3.355 (3)	146.
C16—H16A···O5^ii^	0.96	2.57	3.421 (3)	148.
C16—H16C···S^ii^	0.96	2.87	3.727 (3)	149.

Symmetry codes: (i) −*x*, −*y*+1, −*z*; (ii) −*x*+1, −*y*+1, −*z*.
